# An Update to Biomechanical and Biochemical Principles of Retinal Injury in Child Abuse

**DOI:** 10.3390/children11050586

**Published:** 2024-05-12

**Authors:** Kourosh Shahraki, Donny W. Suh

**Affiliations:** Department of Ophthalmology, Gavin Herbert Eye Institute, University of California Irvine School of Medicine, Irvine, CA 92697, USA; kourosh.shahyar@gmail.com

**Keywords:** abusive head trauma, shaken baby syndrome, retinal hemorrhage, retinal injury, acceleration–deceleration injury

## Abstract

Abusive head trauma (AHT) is an extreme form of physical child abuse, a subset of which is shaken baby syndrome (SBS). While traumatic injury in children is most readily observed as marks of contusion on the body, AHT/SBS may result in internal injuries that can put the life of the child in danger. One pivotal sign associated with AHT/SBS that cannot be spotted with the naked eye is retinal injury (RI), an early sign of which is retinal hemorrhage (RH) in cases with rupture of the retinal vasculature. If not addressed, RI can lead to irreversible outcomes, such as visual loss. It is widely assumed that the major cause of RI is acceleration–deceleration forces that are repeatedly imposed on the patient during abusive shaking. Still, due to the controversial nature of this type of injury, few investigations have ever sought to delve into its biomechanical and/or biochemical features using realistic models. As such, our knowledge regarding AHT-/SBS-induced RI is significantly lacking. In this mini-review, we aim to provide an up-to-date account of the traumatology of AHT-/SBS-induced RI, as well as its biomechanical and biochemical features, while focusing on some of the experimental models that have been developed in recent years for studying retinal hemorrhage in the context of AHT/SBS.

## 1. Abusive Head Trauma and Shaken Baby Syndrome

Abusive head trauma (AHT) in pediatrics, commonly known as shaken baby syndrome (SBS), predominantly affects infants and young children, leading to brain injuries. The injury can result from shaking, blunt impact, or a combination of both, causing neurological damage. This form of child abuse is the most perilous and lethal. Typically occurring in children under 5 years old, abusive head trauma involves injury to the intracranial contents or skull due to violent shaking or blunt impact, with outcomes ranging from complete recovery to severe brain damage and death. Brain and head injuries stand as the leading cause of traumatic death in children under 2 years old. Early diagnosis is crucial but often challenging, with responsible individuals frequently being evasive. Recognition by health professionals is hindered by the lack of external signs, making caregiver education and health provider training essential to prevent accidental abusive head trauma and shaken baby syndrome. Proactive mental health care emerges as the most effective strategy to diminish child abuse, given its long-term financial and medical repercussions for survivors [1].

Though they are often used interchangeably, AHT and SBS can be discerned from one another based on the pattern of injury, which can be described accordingly.

When suspecting a child to be a case of AHT, certain recommendations should be considered, which include the following [2,3]:

- Damage to the contents of the skull or intracranial region in infants or children under the age of 5, commonly caused by forceful shaking or blunt impact.
- The Centers for Disease Control and Prevention (CDC) and the American Academy of Pediatrics advise adopting the term “abusive head trauma” to encompass injuries arising from various conditions, such as shaking, blunt impact, suffocation, and strangulation.
- “Injuries resulting from dropping and throwing a child are also considered within the category of “abusive head trauma”. The term refers to the nature of the injury rather than the specific mechanism”.
- The legal implications of “abusive head injury” are tied to the precise method of harm inflicted. Diagnosing a child with “shaken baby syndrome” can limit the admissibility of evidence related to alternative injuries, potentially complicating legal proceedings. The majority of abusive head trauma cases are typically under a year old, frequently falling within the age range of 3 to 8 months. However, these injuries can extend to children up to 5 years of age.
- Abusive head trauma stands as the leading cause of fatality and impairment in infants and young children affected by child abuse. Child abuse has been pinpointed as the predominant cause of brain injuries in one-fourth of children aged two and older.

In the case of SBS, other recommendations should be considered that include the following [2,3]:

- Abusive head trauma characterized by a distinct pattern of injuries may manifest with retinal hemorrhages and consistent patterns of brain injury. Additionally, fractures of ribs and the ends of long bones are commonly observed.
- The term “shaken baby syndrome” is employed to characterize symptoms of brain injury that align with the forceful shaking of an infant or small child. These injuries typically involve unilateral or bilateral subdural hemorrhage, bilateral retinal hemorrhages, and diffuse brain injury. Although children can sustain injuries from shaking alone, there is frequently accompanying evidence of blunt trauma. Therefore, a more comprehensive term, “shaken impact syndrome”, may be utilized to encompass both shaking and associated blunt trauma.
- The SBS triad comprises encephalopathy accompanied by a subdural hematoma and retinal hemorrhage. However, the diagnosis of pediatric abusive head trauma requires a comprehensive medical examination and thorough testing. Relying solely on the presence of these three findings is not sufficient for an accurate diagnosis.

## 2. Retinal Hemorrhage in Children

Abusive head trauma (AHT) involving acceleration−deceleration injury in children, particularly those under 5 years old, may result in retinal hemorrhage (RH). One important consideration that should be made in the case of AHT-induced retinal hemorrhage is that the injury may not necessarily be the result of blunt impact, since retinal hemorrhage can in fact ensue acceleration−deceleration force or a “shake” alone that does not usually leave a physical mark on the body. As such, it is suggested to rule out other potential causes of retinal hemorrhage first before making a diagnosis of AHT. One potential cause of retinal hemorrhage is coagulopathy, which encompasses diverse pathologies that prevent timely clotting and cessation of bleeding. Still, according to a recently published systematic review, even the presence of an underlying coagulopathy cannot be considered a concrete reason to rule out the possibility of AHT, since certain pathologic findings including retinoschisis or retinal folds are mostly attributed to abusive trauma and not coagulopathies [1].

**Figure 1:**
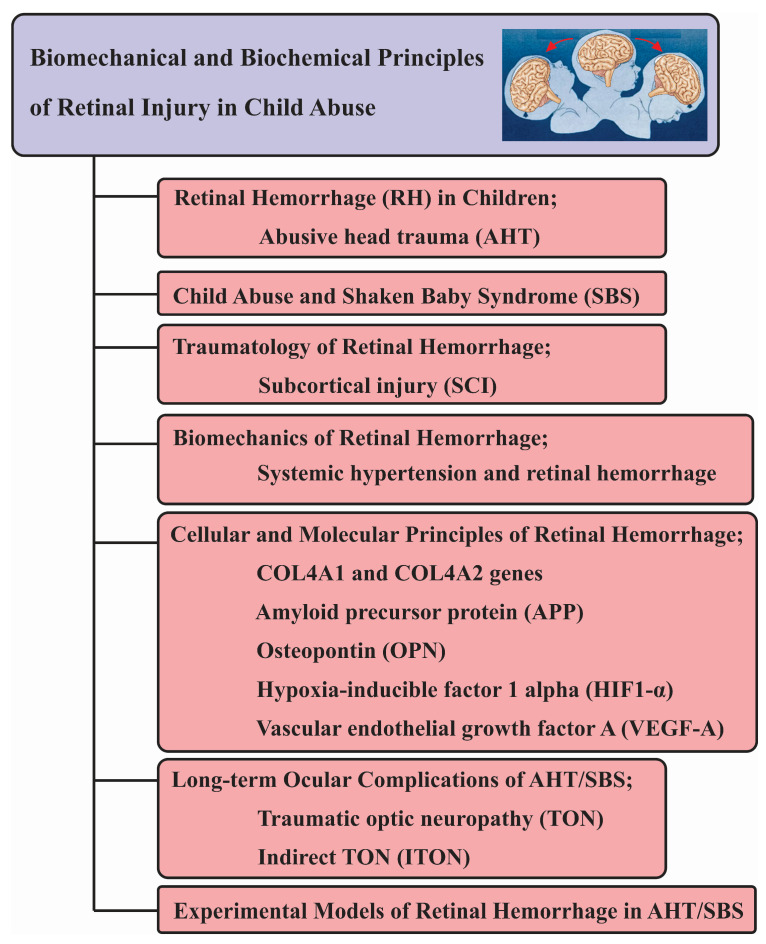
Graphical abstract.

While studies have proposed a number of predictive factors for retinal hemorrhage, traumatic signs on the skin or fractures in areas other than the skull does not stand among them, hence the prerequisite for further examinations. However, subdural hemorrhage, injury to the posterior region of the head or occipital lobe, a Glasgow coma score (GCS) below 15, convulsive episodes and vomiting have been suggested to be fairly reliable predictors of retinal hemorrhage [4,5]. Nevertheless, clinicians are expected to be wary of pitfalls with regard to this delicate issue, as the co-occurrence of subdural and retinal hemorrhage cannot irrefutably indicate AHT, when there is precedent that such clinical picture might be suggestive of life-threatening conditions such as cerebral venous sinus thrombosis, which falls within the category of coagulopathies [6]. In the case of younger children or infants, the situation is substantially more complicated because retinal hemorrhage in infants often occurs secondary to an underlying intracranial pathology of unknown origin [7]. In addition to this, one should not overlook the possibility that younger infants or newborns may exhibit non-traumatic macular hemorrhage that constitutes a non-negligible proportion of eye diseases that warrant referral to an ophthalmologist provided that foveal impingement is present as well [8]. As a matter of fact, neonatal retinal hemorrhage is said to be the most frequent pathology of ocular fundus among neonates with a prevalence slightly lower than 25% in Eastern Asia [9]. Lastly, children with retinal hemorrhage as a result of AHT are at increased odds of death by over 100% as indicated by a national analysis in the US [10] (Figure 1).

## 3. Retinal Hemorrhage in Abusive vs. Non-Abusive Head Trauma

In 2013, a systematic review aimed to examine retinal hemorrhage (RH) and its distinguishing characteristics in cases of abusive head trauma (AHT) versus non-abusive head trauma (nAHT) to enhance the diagnostic accuracy of RH [11]. Conducted meticulously, a systematic review spanning from 1950 to 2009 adhered to rigorous appraisal standards. Stringent inclusion criteria mandated clear confirmation of the trauma’s cause, detailed ophthalmologist examinations, and subjects under 11 years old. Exclusions comprised post mortem data, eye diseases, and poorly conducted exams. Employing multivariate logistic regression, the analysis sought to determine odds ratios (OR) and probabilities specific to AHT. Among the 62 studies analyzed, 13 provided prevalence data for 998 children, with 504 diagnosed with AHT. RHs were substantially more prevalent in AHT cases, constituting 78% compared to only 5% in nAHT cases. Impressively, the likelihood of RH being attributed to AHT surged significantly, with an OR of 14.7 (95% confidence intervals 6.39, 33.62), indicating a remarkable 91% probability of abuse. Additionally, RHs were bilateral in 83% of AHT cases versus a mere 8.3% in nAHT incidents. Noteworthy differences emerged: RHs in AHT cases were numerous, spanning all retinal layers, and often extending to the periphery, while in nAHT, they were sporadic, unilateral, and primarily located in the posterior pole, with only 10% extending to the periphery. Despite certain RH patterns being more common in AHT, no single retinal sign was definitive of abusive injury [11]. These findings highlight the rarity of RH in accidental trauma and stress the need for meticulous differential diagnosis, especially in pediatric head trauma cases where RHs are implicated.

**RH in AHT**: Severe retinal hemorrhages (RHs) are a prevalent finding in infants diagnosed with abusive head trauma (AHT), with as many as two-thirds exhibiting distinct characteristics: bilaterality, involvement of multiple retinal layers, numerousness, and extension to the ora serrata [12,13,14,15]. Notably, unilateral severe RH has also been reported [11]. Moreover, the presence of retinoschisis cavities, primarily associated with AHT, proves highly sensitive for diagnosing this form of trauma [12,16]. Evidence suggests that vitreoretinal traction, resulting from the firm attachment of the vitreous body to specific retinal regions like the vascular arcades, posterior pole, and ora serrata in infants and young children contributes significantly to the development of severe RH observed in AHT cases. This traction, exerted during acceleration–deceleration forces in head injury events, leads to tissue stress, hemorrhage, or physical separation of retinal layers, highlighting the unique pattern of retinal injury in AHT distinct from other conditions [16]. A comprehensive study evaluating a refined web-based tool for documenting RH characteristics in suspected AHT demonstrated good to very good agreement among pediatric ophthalmologists, emphasizing specific attention to various retinal zones [12]. Additionally, a prospective study differentiating between accidental and abusive head injuries in infants under 24 months underscored the diagnostic importance of retinal hemorrhages frequently observed in abusive head injuries and often presenting with bilateral involvement and extension to the retinal periphery [13]. Further research on critically ill children revealed that while RHs were common, severe multilayered RHs resembling those in AHT were rare and usually associated with specific clinical contexts, such as fatal accidental trauma or severe coagulopathy [14]. Additionally, the correlation between retinal hemorrhage distribution and regional cerebral parenchymal injury patterns enhances the diagnostic significance of RH in inflicted head injury cases [17]. Accurate diagnosis heavily relies on proper documentation and detailed description of RH, which serve as cardinal manifestations of AHT [16].

**RH in nAHT:** In nAHT cases, RHs typically stem from a single blunt force impact, like a simple short fall, with hemorrhages often being limited in number, unilateral, and mainly confined to the posterior pole [18]. Nevertheless, severe RHs, including retinoschisis, have been documented in high-energy injuries, such as fatal motor vehicle crashes or falls exceeding ten feet, consistent with the trauma severity experienced [19,20]. While increased intracranial pressure may contribute to RH development in nAHT, such instances are rare and tend to be nonspecific, with only a few hemorrhages localized to the posterior pole [18]. Comparative research on RH patterns between AHT and nAHT highlights the diagnostic importance of these hemorrhages. A systematic review indicates a significantly higher RH prevalence in AHT cases compared to nAHT, with bilateral involvement more prevalent in AHT [11]. Moreover, RHs in AHT cases exhibit distinctive features, including numerous hemorrhages distributed across all retinal layers and extending into the periphery, contrasting with the unilateral, sparse, and posteriorly confined hemorrhages seen in nAHT [11]. Investigations into ophthalmic findings in infants with sparse retinal hemorrhages associated with isolated epidural hematomas offer further insights, indicating no suspicion of non-accidental trauma in such cases [18]. Hence, comprehensive evaluations and meticulous documentation are crucial in distinguishing between AHT and nAHT, considering the unique RH characteristics observed in each scenario.

## 4. Shaken Baby Syndrome from the Ophthalmologists’ Perspective

Shaken baby syndrome (SBS) is a variant of child abuse that occurs most often in response to incessant crying of the infant by an irritated parent or guardian [21]. Depending on the magnitude and duration of force being imposed on the child, SBS may instigate hemorrhagic injuries to intracranial structures as well as the retina. Whether an infant may fall victim to SBS is determined based on several risk factors including, but not limited to, premature birth (associated with a more difficult upbringing) and neuropsychiatric disorders in parents [22]. Generally, SBS is a rare condition, and because of this, studies focusing on this form of maltreatment are considerably limited with regard to their sample size. For example, a limited investigation conducted on SBS involving a series of cases from 2004 to 2020 in Portugal had only managed to include eight patients, whose mean age was 102 ± 69 days, indicating that SBS may occur at any time from 1 to 3 months after birth. Conversely, according to a preliminary study on a series of eight children, the mean age of the perpetrator (mother, father or even babysitter) was reported to be 23 ± 4 years [23], suggesting that SBS might be more likely to take place with inexperienced or young guardians [24].

The overall clinical presentation of SBS may include crying, lethargy, hypotonia, and bone fractures in the case of abusive head trauma. In younger patients, concordant abnormal findings regarding the fontanelles such as bulging can be considered an alarming sign. On a more holistic level, most patients may demonstrate hemorrhage either at the subdural region or retina [23]. Retinal hemorrhage can be as common as 100%, according to a report describing findings from a Swedish cohort of SBS cases [25]. Similarly, Wright et al. (2021), who explored visual complications in a series of 44 children (median age: 16 weeks), reported a high prevalence of subdural hemorrhage (98%) and retinal hemorrhage (93%) in the population under investigation [26]. Moskwa et al. [27] conducted an analysis of ophthalmologic reports encompassing 133 patients within their institution, employing pharmacologically induced mydriasis and posterior segment photography. This examination provided valuable insights for further exploration. For instance, the laterality of retinal lesions seemed to correlate with the age at diagnosis, with subjects over 6 months more frequently exhibiting bilateral findings compared to those under 6 months. These differences are primarily attributable to anatomical factors, challenging the accuracy of proposals put forth by previous authors who did not consider the infant’s age at the time of diagnosis [28,29]. Furthermore, there was no apparent association between the laterality of intracranial lesions and ocular lesions. Importantly, the limited diagnostic value of bilateral findings compared to unilateral ones became evident. Barth et al. in their case report emphasized the possibility of unilateral retinal hemorrhages in cases of AHT, particularly if the traumatic insult is localized on a specific side. Notably, both reported cases involved infants under 3 months of age, supporting the hypothesis that an age exceeding 6 months is a predisposing factor for the onset of ocular injuries. Recent investigations have also suggested an inverse association between the likelihood of retinal hemorrhage and age because children under the age of 2 years are more likely to develop RH as a result of AHT [30]. Table 1 is a list of ocular manifestations of SBS.

When evaluating retinal lesions in patients suspected to have SBS, several considerations should be made, an important one of which is to distinguish SBS-induced retinal hemorrhage from RH of other causes, such as Terson’s syndrome and Purtscher’s retinopathy. Characteristic cues include lateralization, quantity, and localization of retinal hemorrhages, which are observed as numerous hemorrhagic spots in both eyes that are distributed across all layers of the retina, extending from the central compartment to the periphery [44]. It is highly recommended to perform a thorough eye examination within 48 h of the admission of any pediatric case suspected to have AHT or SBS. Despite this, nearly 19% of SBS cases are faced with a delay in primary fundoscopic examination [45]. The majority of these patients whose condition is complicated by delays, however, are usually older and present with isolated cranial fractures, which could partly explain the reason for postponement of the retinal examination [46]. Nevertheless, it is still imperative to perform initial fundoscopy since the risk of SBS is children with RH is three times more likely [47].

## 5. Links between Intracranial Injury and Retinal Injury in Infantile AHT

Retinal hemorrhages stemming from abusive head trauma (AHT) present with discernible ophthalmologic attributes. These hemorrhages often manifest bilaterally, displaying a multilayered involvement that encompasses both the inner and outer retinal layers. Predominantly situated in the peripheral regions of the retina, they notably spare the macula. The coexistence of retinal folds or tears serves as additional indicators, providing insights into the gravity of the traumatic impact. Furthermore, AHT-related retinal hemorrhages exhibit a heightened prevalence and broader distribution compared to those associated with accidental trauma. The specific ophthalmologic characteristics, encompassing size, distribution, and structural nuances, alongside their correlation with other clinical observations, assume paramount importance in the diagnostic scrutiny of abusive head trauma cases. Acknowledging these distinctive ophthalmologic features is essential for healthcare professionals and forensic experts in accurately interpreting retinal hemorrhages as potential markers of non-accidental trauma in infants and young children [1].

Several clinically important correlations have been proposed that are implicated in SBS or AHT. Based on the findings of Even et al. in 2021, which were derived from an investigation on infants with traumatic brain injury from 2011 to 2021, subcortical injury (SCI) is substantially associated with AHT. On average, SCI predicts a roughly 750% increased risk of AHT in children. To a slightly lesser (but still significantly high) extent, SCI is also correlated with RH and rib fractures. As a matter of fact, children with SCI are six times more likely to have RH, suggesting that subcortical injuries may warrant a careful fundoscopic examination and vice versa [48]. Another study by Kato et al. on a cohort of 452 Japanese children concluded that SDH and cerebral edema might predict RH, with markedly high odds ratios of 23.41 and 5.46, respectively [49]. These correlations, however, may differ from one population to another. For example, cerebral edema was not found to bear any association of statistical significance with AHT, as observed with a series of Australian patients in late 2022 [50]. Regardless of racial differences with regard to pathologic features, clinical diagnosis of SBS or AHT is suggested to be made based on observation of the triad, which includes three cardinal signs: (1) the presence of SDH or any other form of fluid collection; (2) an altered level of consciousness at any time starting from the onset of injury to hospitalization; and (3) the presence of hemorrhage across a large area of the retinal surface that can be spotted at the ora serrata as well. As reported by Hymel et al. in 2022, the triad (not the Ontario triad) confers a sensitivity and specificity of 51% and 96% for AHT, respectively [32]. It should be noted that alongside the conventional triad mentioned here, a slightly different triad known as the Ontario triad is preferred by some ophthalmologists. The important difference between the two is that the latter does not take into account the lack of consciousness, and instead includes a different third criterion, which is “absence of any signs of impact”. While the Ontario triad is superior in terms of specificity (100%), it falls behind with regard to sensitivity (32%) [32].

Subcortical brain injury as a result of AHT is another type of injury that should be carefully appraised in the context of retinal injury. This is due to the fact that subcortical injury could lead to impairment of the subcortical visual circuitry, which includes several pathways that are responsible for relaying visual signals. The retino-tectal or retino-collicular pathway is an important component of this circuitry that regulates postural stability through shifting eye position in a reflexive manner. The accessory optic system is another important constituent of the subcortical visual circuitry and is tasked with stabilizing retinal images during movements of the head [51]. Subcortical injury may occur following AHT in children. In 2022, an investigation by Even et al. on 973 children (aged < 3) with acute traumatic brain injury referring to 18 PICUs in the US reported a prevalence of 25.38% for subcortical injury, which was higher than cortical brain injury (17.47%), suggesting that subcortical injury could occur with a rough likelihood of 25% upon traumatic brain injury. An interesting aspect of this study was that, according to its results, children with AHT were at significantly higher odds (OR: 8.41) of developing subcortical injury, indicating the importance of this type of brain injury in examination procedures [48]. 

## 6. Biomechanics of Retinal Hemorrhage

Considering the “shaking” element in SBS, the main cause of retinal injuries in such cases is the indirect impact associated with the force being imposed on the patient. Oftentimes, the causal force is of acceleration−deceleration type, which is characterized by sudden and unrestricted movements that result in spatial and temporal compression of the target tissue (retina in this case) as a consequence of shear strains [52,53]. Traction is another term that can be used to describe the pulling-induced nature of these injuries. It is assumed that vitreoretinal traction might be involved in the pathogenesis of retinal hemorrhage associated with SBS, as it is known to facilitate the formation of perimacular folds [29]. One potential culprit is thought to be the increased intracranial pressure following AHT. This has been confirmed in part by Thilbin et al., who explored a population of 148 infants with suspected maltreatment, i.e., abusive trauma to the head. They observed intracranial pathology in 97% of infants who had presented with retinal hemorrhage, which was significantly higher than that of the patients without retinal hemorrhage equaling 13% [7]. In 2021, investigators from the US developed a finite element analysis (FEM)-based model to predict the distributional effects of tractional forces on patterns of retinal hemorrhage occurring in SBS or AHT. Due to the controversial nature of real-life models with regard to AHT, computer simulations are usually preferred. Having applied various pressures roughly ranging from 3 to 16 kPa (as per the simulations mentioned in the article) on their virtual model, Suh et al. observed that the peripheral region of the retina had been affected the most, followed by the posterior retinal pole. These observations were consistent with the characteristic features of RH that are present in patients with SBS, considering that the forces applied to all three retinal layers (e.g., pre-, intra-, and subretinal) were similar in magnitude [54]. The greatest limitation of FEM, however, is that it is a method used for computer-based (and not real-life) simulation, which renders its results subject to differences compared with real life [55]. It is speculated that more frequent exposure to an acceleration−deceleration force may not be sufficient to induce RH. A bona fide example in this case is the family of *Picidae* or woodpeckers, which are known to withstand accelerations as high as 1000 g (gravity) while pecking without developing retinal hemorrhage. The astonishing principles governing the durability of retinal layers was explored in 2020 by a group of scientists from China, who had developed finite elements models of both human and woodpecker eyes to this end. This study identified several factors in the woodpecker eye that contributed to the resistance of retinal structures against acceleration−deceleration injuries that included lack of vitreoretinal attachment and scleral ossification, which contradict the normal anatomy of the human eye [56]. Since vitreoretinal attachment depends of a series of biochemical factors, such as collagen fibers, there might be certain associations among RH, vitreoretinal attachment, and deregulation of anchoring fibrils [57].

## 7. Ocular Hypertension and Retinal Hemorrhage

While the structural basis of the inner eye plays a significant part in the occurrence of RH, a growing number of investigations have continued to highlight the role of intraocular pressure (IOP), as well as intracranial pressure on a larger scale, in AHT-induced retinal hemorrhage. It is speculated that in addition to shaking forces associated with SBS, hypertension might also be implicated in the emergence of RH. This could be a rational explanation for the exclusivity of RH with regard to AHT or SBS, considering that it rarely occurs in accidental head injuries, as opposed to non-accidental incidents. This has been tested several times using experimental models such as a porcine eye model, which was developed by Umstead et al. in 2020. The aim was to induce intraocular hypertension through pressurizing the eye by means of fluid retention. According to the findings, hypertension or increased IOP alone was not accompanied by RH, though when combined with shaking forces, hypertension managed to induce RH in the experimental eye model [58], suggesting that children with increased IOP can be more susceptible to RH following non-accidental heal trauma. Additionally, there is also a direct component to the correlation between IOP and trauma since trauma itself can be a causal factor for increased IOP or glaucoma in children [59]. From a biomechanical perspective, an IOP of 24 mmHg and above is considered a risk factor for childhood glaucoma [60], while an IOP of 21 mmHg is considered the upper limit of normal intraocular pressure. In 2019, an investigation led by Kalamkar et al. sought to explore the demographic features and clinical outcomes of post-traumatic elevated IOP in a cohort of Indian children aged < 16, reporting a prevalence of 15.6% for elevated IOP after trauma characterized by a mean pressure of 29.8 ± 6.3 mmHg [61].

## 8. Cellular and Molecular Markers of Retinal Hemorrhage

**Figure 2:**
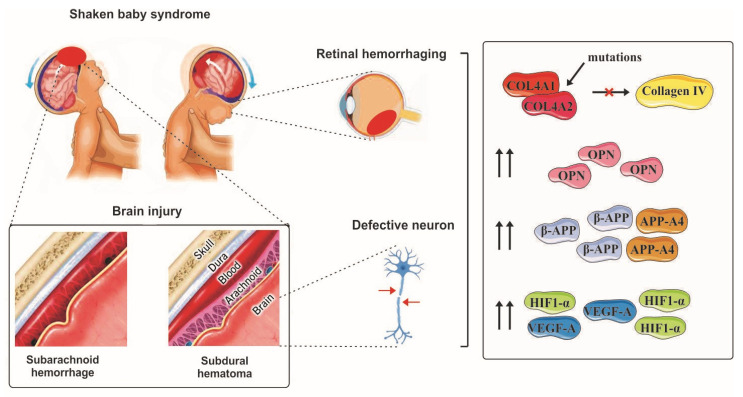
Schematic view of molecular change in AHT. The image shows shaken baby syndrome effects on brain, eye, and neurons: It explains structural and molecular impacts.

In contrast to the gross pathologic features of retinal hemorrhage, very little, if anything, is known regarding molecular mechanisms that might play a potential part in AHT/SBS-related RH. Hopefully, this knowledge gap is actively being filled by ongoing studies. In fact, what we already know about the molecular underpinnings of RH has been published in the last few years (Figure 2). More often than not, mutations in genes coding for different types of collagens have been implicated in cerebral and retinal angiopathies characterized by hemorrhage. For instance, cerebral hemorrhage in newborns and infants has been attributed in part to mutations in COL4A1 and COL4A2 genes that encode the α1 and α2 subunits of type IV collagen, respectively. Such mutations predispose children to developing retinal arteriolar tortuosity, which is a known risk factor for recurrent retinal hemorrhage [62,63]. In addition to this, one recent study indicated the expression of type VII collagen at the vitreoretinal interface (VRI) in humans, which is thought to contribute to the vitreoretinal attachment due to its anchoring function [57]. It should be noted, however, that collagen changes do not directly predispose children to retinal hemorrhage in the context of AHT.

Another predisposing factor could be the amyloid precursor protein (APP), which is a conserved transmembrane protein and widely implicated in the neural tissue. While studies on APP in the context of AHT/SBS are not as extensive as those concerned with collagen, there is sufficient information to suggest a role for APP as a molecular element in hemorrhagic complications associated with AHT/SBS. In 2015, an investigation by a group of scientists from the Netherlands on 37 infants’ AHT revealed that children with retinal hemorrhage were significantly more likely (OR: 11.4) to express beta-APP (β-APP) in their retinal tissue compared to those without RH [64]. More recently, in 2022, a retrospective case–control study by Minckler et al. on 72 eyes from AHT cases confirmed accumulation of AAP-A4 in the retina and lamina cribrosa using immunohistochemistry [65]. The importance of amyloid precursors and amyloid itself, particularly amyloid beta (Aβ), stems from the hazardous effects of these protein complexes on the retina since Aβ can cause cellular damage to the retinal pigment epithelium (RPE) and activate the inflammatory response by positively regulating the NLRP3 inflammasome pathway within the retinal tissue [66]. More recent investigations have attributed the pro-inflammatory effects of Aβ in part to the poly(ADP-ribose) polymerase (PARP1)/SIRT1 pathway in RPE cells. Based on these studies, accumulation of Aβ1-42 in retinal cells can result in activation of PARP1 and subsequent repression of sirtuin 1 (SIRT1), ultimately leading to increased expression of caspase-3 and caspase-9, hence accelerated apoptosis [67]. The deleterious effects of amyloid aggregates are not restricted to epithelial pigment cells, as these complexes can also negatively affect Muller cells or retinal gliocytes, which are involved in neuroprotection and immunity [68].

Besides collagen and AAP, osteopontin (OPN) has also been proposed as a biomarker for diagnosis of AHT in cases presenting with SDH or RH. OPN is a sialoprotein and an inherent component of the mineralized extracellular matrix, which is found in the skeletal tissue. The diagnostic value of OPN was confirmed by Blackwell et al. in 2020 when they surveyed the serum level of OPN in 77 children (aged ≤ 4) who were suspected to have experienced AHT. They reported a significant rise in OPN levels in children who had signs of intracranial hemorrhage, which coincided with an increased release of OPN from microglia following brain injury [69]. Microglia are equivalents of macrophages in the nervous system and serve important purposes during normal development, which is an active process in children. These cells regulate the population astrocytes, a subtype of glial cells that are present in large numbers as an integral component of the neural tissue including the retina as well. During development, astrocytes are engulfed and phagocytosed by microglia as a natural process. This occurs in addition to apoptotic cell death, which is mediated by astrocytes themselves. Dysregulated activity of microglial cells as a consequence of genetic ablation (or other events) may result in disinhibited proliferation of astrocytes, which is known to predispose individuals to retinal hemorrhage by influencing the retinal vasculature [70]. Involvement of OPN in different subtypes of retinal injury has been documented over the years by several investigations. Recently, in 2023, Zhao et al. found that OPN is upregulated in retinal ganglion cells (RGCs) under glaucomatous conditions and presumably promotes the resiliency of these cells, making RGCs adapt to their new condition [71]. Based on the current evidence, it appears that upregulation of OPN following acute retinal injury might be a defense mechanism, as OPN is known to improve cell survival and migration following cell damage. In their 2018 investigation, Ruzafa et al. developed a murine model of retinal aging in which the OPN gene was knocked out. The aim of this study was to confirm if OPN might have any temporal effects on the population of RGCs and Muller cells. They found that mice lacking OPN were significantly deficient in terms of RGC density, as they observed that the inability to express OPN was associated with a 25% and 60% decrease in the density of RGCs in 3- and 20-month-old mice, respectively. In a way similar to RGCs, astrocytes were also observed to have been negatively influenced by OPN deficiency, suggesting a neuroprotective role for OPN [72]. OPN also plays a role in our daily lives by protecting us against the blue light being emitted from screens, as evidenced by Chang et al. in 2016 in a series of mice that were exposed to blue light-emitting diodes and were later found to have increased expression of OPN in their retinal tissue, indicating that OPN could be considered a marker of retinal degeneration [73]. These findings are in agreement with the results of an earlier study by Duan et al. (2015), who successfully managed to induce regeneration of RGCs by simultaneously treating murine RGCs with OPN and insulin-like growth factor 1 (IGF-1) [74]. Although OPN may appear to be of potential diagnostic value, one should not overlook the fact that the findings mentioned here are totally based on murine models and do not include data on humans.

## 9. Long-Term Ocular Complications of AHT/SBS

While retinal hemorrhage, especially subretinal hemorrhage, is the immediate response of the retina to injury-induing forces, ocular complications of AHT/SBS are known to stretch beyond acute bleeding. Hemorrhage itself is associated with functional and morphological disruptions in the vascular wall, which may result in impaired circulation at the injury site and optic nerve pallor [75]. This in turn can bring about progressive atrophy and the death of neuronal cells, particularly retinal ganglion cells (RGCs), which are responsible for providing a neuronal circuit between the retinal input and visual processing centers in the brain. This was confirmed in part through an investigational attempt by Li et al. in 2022, who developed an animal model of traumatic optic neuropathy (TON) to explore the molecular changes associated with optic nerve crush injury. They found that blunt trauma directed at the eye was followed by oxidative stress and apoptotic death of RGCs, resulting in a diminished population of these bridging cells, which was molecularly characterized by marked downregulation of miR-181d-5p [76] coupled with increased accumulation of amyloid precursor protein [65], with the latter being reported by another study. One step beyond the molecular infrastructure, at the cellular level, the pathologic alterations in the population of RGCs were confirmed by Ma et al. in 2022, who examined a series of patients with indirect TON (ITON) using optic coherence tomography angiography (OCTA), highlighting significant thinning of the ganglion cell complex (GCC) [77]. The thinning effect and perpetual atrophy of the retina may persist for up to 35 years after the onset of injury. This can be visualized by immunohistochemical staining of ganglion cells using FluoroJade-C, a high positivity of which is associated with neurodegeneration [78]. Still, the current evidence is highly marginal and does not suggest direct involvement of ganglion cell injury and optic neuropathy in AHT-associated RH.

RGC atrophy and death, in the context of traumatic injury, is further accelerated by the generation of reactive oxygen species (ROS) as a component of oxidative stress [79]. In 2021, Balsak and Deveci provided evidence for this by measuring the levels of malondialdehyde (MDA) and myeloperoxidase (MPO), which are indicators of oxidative stress. They found that both markers were upregulated as a result of retinal damage secondary to traumatic head injury [80]. Increased oxidative stress is further supported by the findings of Hetzer et al., who suggested upregulation of stress markers in the endoplasmic reticulum (ER) of retinal cells after TON, which was correlated with gliosis and neurodegeneration [81].

Epigenetic alterations, as long-term sequelae of traumatic retinal injury, are expected, despite the limited evidence. In late 2022, Xu et al., explored the effects of stress-related aging on visual function in young (3-month-old) and old (18-month-old) mice. To determine potential correlations between aging and DNA methylation, they used a PanTissue mouse clock, which is a form of epigenetic clock. The resulting genome-wide analysis revealed increased methylation at 61 cytosine–phosphate–guanosine (CpG) sites, with marked demethylation at over 1200 CpG sites, confirming the epigenetic basis of traumatic retinal pathology [82]. Another investigation by Gupta et al. in 2019 on the genome methylation profile of RGCs following traumatic brain injury indicated hypermethylation of the ninth lysine residue on histone H3 proteins (H3K9) as a result of increased G9a enzymatic activity (a histone methyltransferase), which coincided with accentuated oxidative stress [83]. While the current evidence is inconclusive, the process of aging, as of recently, has elicited interest among investigators aiming to simulate this process in the retina by means of different modeling systems, especially finite element analysis (FEM). In 2022, Khoobyar et al. explored the effects of aging-related partial liquefaction on vitreous humor using an FEM model, reporting certain degrees of shift in pressure-driven flow across the vitreous humor (simulated in this study with a porous medium), which could be a consequential finding regarding retinal integrity upon trauma-induced pressure [84]. In the same year, Shukla et al. performed a FEM-based investigation on age-related retinal detachment, with an emphasis on aging [85]. Retinal detachment is an emergency condition in which the delicate retinal tissue is pulled away from its underlying supportive tissue. While retinal detachment is not a frequent complication of AHT in children, it might still occur under certain circumstances, as in the two infantile cases reported by Sornalingam et al. that had been referred to the hospital with retinal detachment following non-accidental trauma to the eye [86]. In their study, Shukla et al. adopted the FEM method to estimate the response of the human eye (including retina) to scleral buckle surgery, which is performed in the case of retinal detachment. As part of their simulation, they observed a maximum displacement of 3.3481 mm and a stress of 35.9 KPa at 0.7 MPa pressure at the sight of retinal detachment [85]. In the context of aging-related retinal changes, FEM has also been augmented with methods such as discontinuous Galerkin for simulation of cellular alterations observed on optical coherence tomography of the human retina [87].

## 10. Less Explored Risk Factors of Retinal Hemorrhage in the Context of AHT

In addition to the associations discussed thus far, other factors, including intrathoracic pressure, vascular fragility, and anemia, might contribute to the emergence of RH in children that are exposed to non-accidental trauma. The connection between retinal hemorrhage and intrathoracic pressure involves intricate physiological interactions. Elevated intrathoracic pressure, as observed during instances of forceful coughing or vomiting, can induce alterations in blood flow and pressure within the retinal blood vessels. These changes may contribute to the occurrence of retinal hemorrhages. Conditions associated with heightened intrathoracic pressure, such as specific respiratory or cardiovascular disorders, may further increase the susceptibility to retinal vascular modifications. Appreciating the nuanced interplay between these factors is essential in both clinical and forensic scenarios, as retinal hemorrhages can serve as potential indicators of underlying health issues or, in cases of abusive head trauma, significant forensic evidence. However, it is crucial to approach these connections with a comprehensive understanding of the individual’s unique medical history and circumstances to ensure accurate interpretation [88]. Understanding the relationship between vascular fragility in neonates and the occurrence of retinal hemorrhages holds significant implications, particularly in the context of abusive head trauma (AHT). Neonates, with their developing vascular systems, possess more delicate retinal blood vessels, rendering them susceptible to spontaneous bleeding. This inherent fragility becomes especially relevant when considering external forces such as those associated with AHT, where forceful shaking or blunt impact can amplify the risk of traumatic injury to the retinal vasculature, leading to retinal hemorrhages. Acknowledging the increased vulnerability of neonatal blood vessels is paramount for clinicians and forensic experts alike, providing crucial insights into the potential etiology of retinal injuries, including non-accidental trauma. This awareness underscores the necessity of considering neonatal physiology when evaluating cases involving retinal hemorrhage, both medically and forensically [89,90]. The interconnection between anemia and retinal hemorrhage in infants or children introduces a nuanced consideration, particularly in the realm of medical and forensic scrutiny, such as in cases of abusive head trauma (AHT). Anemia, characterized by diminished oxygen-carrying capacity in the blood, can potentially influence blood viscosity and flow dynamics. In the context of AHT, where forceful shaking or blunt impact may lead to traumatic injuries, the compromised oxygen transport in anemic blood could accentuate the susceptibility of retinal blood vessels. The conjunction of anemia and external forces might elevate the likelihood of retinal hemorrhages. A comprehensive understanding of this association is crucial for healthcare practitioners and forensic professionals, providing a deeper perspective on the interpretation of retinal injuries, particularly in situations involving suspected abuse. This recognition of the interplay between anemia and retinal hemorrhage contributes to a more informed approach in medical assessments and forensic investigations, ensuring a thorough examination of ocular manifestations in infants and children [91].

## 11. Experimental Models of Retinal Hemorrhage in AHT/SBS

Considering the causal factor of AHT/SBS, which in the simplest form is an external force, the condition cannot be deliberately induced in humans for investigational purposes. The deeply rooted controversy associated with AHT/SBS has even prevented us from developing live animal models in the majority of instances, making our forensic understanding of AHT/SBS exceedingly limited. This issue, however, has been addressed to some extent with the advent of computer simulations, a popular form of which is known as finite element models (FEM). FEM uses block-like elements to simulate a given object for analysis. The breadth of application of this technique is not merely restricted to AHT/SBS, as it has successfully been adopted for analytic purposes in other “ethically controversial to simulate” eye injuries, such as soccer ball-related eye injury [92]. A recent FEM-based analysis by Lam et al., which was aimed at comparing soccer ball-related retinal trauma with its AHT-related counterpart, found that the trauma caused by soccer ball impact cannot induce the characteristic retinal injury associated with AHT/SBS, since the forces at play are mostly of a linear deceleration type in contrast to the repetitive angular forces associated with AHT/SBS [93].

Several methods have been developed for the purpose of studying the biomechanical profile of RH in the context of AHT/SBS, which are summarized in Table 2. Although data derived from these experimental models are inconclusive, they still have contributed substantially to our understanding of how tractional forces may damage the intricate structure of the inner eye and cause retinal bleeding. Here, the tension pressure applied to each of the given models might appear insignificant, measuring only a few kPa (kilo pascal), though when applied repeatedly, as is the case with SBS, negligible forces may result in subretinal hemorrhage. Based on the findings observed with one such model that was developed by Yoshida et al., in 2014, abusive shakes impose an average pressure of 100 Pa per second to the patient. Given that the retina has an area equal to 1000 mm^2^, a pressure of 100 Pa would indicate that the force being applied would be equal to 0.1 N (newton) or 0.01 kg (10 gr). It is astonishing how repeated shakes with a force of only 10 gr can result in hemorrhage when directly applied to the retina [94]. Nevertheless, development of more realistic models to better study retinal damage following AHT/SBS is still warranted since the findings of FEM in its current state cannot be extrapolated, as FEM is only a computer-based method used for the purpose of simulation. 

## 12. Conclusions

Having reviewed the most cutting-edge evidence relevant to AHT-/SBS-induced RH here, one might conclude that we are still a long way from understanding the biomechanical and biochemical basis of RH in the context of abusive shaking. Despite being recognized as a rare type of abuse, SBS still continues to affect an attention-demanding number of children in the world and can result in life-long visual disability if not examined properly at the time of admission. Certain molecular mechanisms might predispose young children to develop more severe forms of retinal injury in response to acceleration–deceleration forces, which need to be explored more closely both for diagnostic and therapeutic purposes. A deeper understanding of different pathogenic aspects of AHT-/SBS-induced retinal hemorrhage is anticipated to positively contribute to the current protocols developed for clinical management of these unfortunate children. As such, further descriptive research on human participants and investigational attempts on experimental models are encouraged.

## Figures and Tables

**Table 1:** Intracranial and ocular findings associated with AHT/SBS as reported by different studies in the last five years.

Study	Year	Country	Type	Presentation	Tools	Observations	Ref.
**Felez-Moliner et al.**	2022	Spain	Retrospective cohort	Seizure	CCT Fundoscopy	RH (15, 79%) SDH (15, 79%)	[31]
**Hymel et al.**	2022	USA	Case series	Respiratory compromise Circulatory compromise Seizure	CCT Fundoscopy	Bilateral SDH (41, 71.4%) Retinoschisis (12, 21.4%) RH (21, 35.7%)	[32]
**Moskwa et al.**	2022	France	Retrospective cohort	Crying Seizure	CCT Fundoscopy	IVH (9, 6.7%) Macular lesions (11, 8.2%) Papilledema (15, 11.1%) RH (126, 94.4%)	[27]
**Weiss et al.**	2022	USA	Retrospective cohort	Crying Eye contusion Seizure	CCT Fundoscopy	Retinal edema (1512, 59.3%) RH (135, 5.3%) SAH (584, 22.9%) SDH (1856, 72.8%)	[33]
**Eddahabi et al.**	2021	Germany	Case report	Paroxysmal crying Paused breathing Seizure	Fundoscopy	Bilateral RH ILM hemorrhage IVH PVR	[34]
**Flugt et al.**	2021	Denmark	Case series	Contusion Crying Rib fractures Skull fractures	CCT Fundoscopy	Cerebral edema (7, 87.5%) SAH (8, 100%) RH (6, 75%) SDH (6, 75%)	[35]
**Oruç et al.**	2021	Turkey	Retrospective cohort	Cardiopulmonary arrest Seizure	CCT Fundoscopy	Bilateral RH (6, 75%) Unilateral RH (2, 25%) SAH + EDH (1, 12.5%) SAH + SDH (3, 37.5%) SAH + SDH + ICH (4, 50%)	[36]
**Barth et al.**	2020	Germany	Case series	Loss of consciousness Paroxysmal crying Seizure	CCT Fundoscopy	Periorbital petechiae (2, 100%) SDH (2, 100%) Unilateral RH (2, 100%)	[37]
**Donaldson et al.**	2020	Canada	Retrospective cohort	Loss of consciousness Paroxysmal crying Seizure	CCT Fundoscopy MRI	ICH (21, 72.4%) Retinoschisis (5, 18.8%) RH (16, 55.2%)	[38]
**Ksiaa et al.**	2020	Tunisia	Case report	Loss of consciousness Paroxysmal crying	CCT SSOCT	Bilateral SDH Bilaterally poor PLR Bilateral pRH Bilateral iRH Bilateral pMH Bilateral ILM detachment	[39]
**Thilbin et al.**	2020	Sweden	Retrospective cohort	Contusion Crying Rib fractures	CCT MRI X-ray	Bilateral RH (1, 2.7%) Unilateral RH (1, 2.7%)	[40]
**Alnabi et al.**	2019	USA	Case series	Loss of consciousness Respiratory compromise Seizure	CCT Fundoscopy	Bilateral RH (4, 80%) VRT (5, 100%) PMF (5, 100%) ILM detachment (5, 100%)	[29]
**Kelly et al.**	2019	New Zealand	Case report	Acute visual loss Dilated pupils Lethargy	CCT MRI Fundoscopy	Bilateral RH	[41]
**Tripathy et al.**	2018	USA	Case report	Cardiovascular collapse	Fundoscopy MRI	Bilateral SDH ILM hemorrhage Retinoschisis SAH Subhyaloid hemorrhage	[42]
**Wu et al.**	2018	Taiwan	Retrospective cohort	Crying Loss of consciousness Vomiting Respiratory compromise	CCT Fundoscopy	Retinoschisis (20, 26.7%) RH (69, 92%)	[43]

CCT: cerebral computed tomography; EDH: epidural hemorrhage; ICH: intracranial hemorrhage; ILM: internal limiting membrane; IVH: intra-vitreous hemorrhage; iRH: intraretinal hemorrhage; MRI: magnetic resonance imaging; PLR: pupillary light response; PMF: perimacular fold; pMH: premacular hemorrhage; pRH: preretinal hemorrhage; PVR: proliferative vitreoretinal reaction; SAH: subarachnoid hemorrhage; SDH: subdural hemorrhage; SSOCT: swept source optical coherence tomography; VRT: vitreoretinal traction.

**Table 2:** List of models developed for studying ocular injury associated with external stress resembling AHT/SBS.

Study	Year	Country	Experimental Material	Model	Stress Type	Stress Magnitude	Ref.
**Song et al.**	2022	USA	Primate eye Sheep eye	FEM	Linear acceleration Angular acceleration Repetitive shaking	Vitreoretinal detachment: 1–5 kPa Cyclic tension range: 3–16 kP	[95]
**Lam et al.**	2022	USA	−	FEM	Linear acceleration Angular acceleration	Preretinal: 0–1.6 kPa Intraretinal: 0–1.4 kPa Subretinal: 0–1.4 kPa	[93]
**Stray-Pedersen et al.**	2021	Norway	Dummy equivalent of 1-month-old infant	Human perpetrator	Repetitive shaking	Acceleration (X axis): 6.5–36.2 g Acceleration (Y axis): 2.6–11.7 g Acceleration (Z axis): 5.2–44.0 g	[96]
**Nadarasa et al.**	2018	France	Dummy equivalent of 6-week-old infant	FEM	Linear acceleration Rotational acceleration Repetitive shaking	Choroid: 10.7–16 kPa Retina: 1.4–2.0 kPa	[97]
**Yoshida et al.**	2014	Japan	Agar gel (used for modeling vitreous)	FEM	Linear acceleration Rotational acceleration Repetitive shaking	Abusive shake (per cycle): 101 Pa/s Abusive impact: 36 Pa/s	[94]
**Yamazaki et al.**	2014	Japan	Dummy doll	FEM	Cyclic acceleration–deceleration	Shaking (per cycle): 107 Pa/s Fall (per cycle): 60–73 Pa/s	[98]
**FEM: finite element model.**							

## Data Availability

The data presented in this study are available upon request from the corresponding author. The data are not publicly available due to the sensitive nature of the images, which include fundus photos with retinal hemorrhages from Abusive Head Trauma patients.
